# Relationship of systemic, hepatosplanchnic, and microcirculatory perfusion parameters with 6-hour lactate clearance in hyperdynamic septic shock patients: an acute, clinical-physiological, pilot study

**DOI:** 10.1186/2110-5820-2-44

**Published:** 2012-10-15

**Authors:** Glenn Hernandez, Tomas Regueira, Alejandro Bruhn, Ricardo Castro, Maximiliano Rovegno, Andrea Fuentealba, Enrique Veas, Dolores Berrutti, Jorge Florez, Eduardo Kattan, Celeste Martin, Can Ince

**Affiliations:** 1 Department of Translational Physiology, Academic Medical Center, University of Amsterdam, Amsterdam, The Netherlands; 2 Departamento de Medicina Intensiva, Pontificia Universidad Católica de Chile, Marcoleta 367, Santiago, 8320000, Chile; 3 Centro de Tratamiento Intensivo, Hospital de Clínicas, Montevideo, Uruguay

**Keywords:** Septic shock, Hepatosplanchnic perfusion, Lactate, Microcirculation

## Abstract

**Background:**

Recent clinical studies have confirmed the strong prognostic value of persistent hyperlactatemia and delayed lactate clearance in septic shock. Several potential hypoxic and nonhypoxic mechanisms have been associated with persistent hyperlactatemia, but the relative contribution of these factors has not been specifically addressed in comprehensive clinical physiological studies. Our goal was to determine potential hemodynamic and perfusion-related parameters associated with 6-hour lactate clearance in a cohort of hyperdynamic, hyperlactatemic, septic shock patients.

**Methods:**

We conducted an acute clinical physiological pilot study that included 15 hyperdynamic, septic shock patients undergoing aggressive early resuscitation. Several hemodynamic and perfusion-related parameters were measured immediately after preload optimization and 6 hours thereafter, with 6-hour lactate clearance as the main outcome criterion. Evaluated parameters included cardiac index, mixed venous oxygen saturation, capillary refill time and central-to-peripheral temperature difference, thenar tissue oxygen saturation (StO_2_) and its recovery slope after a vascular occlusion test, sublingual microcirculatory assessment, gastric tonometry (pCO_2_ gap), and plasma disappearance rate of indocyanine green (ICG-PDR). Statistical analysis included Wilcoxon and Mann–Whitney tests.

**Results:**

Five patients presented a 6-hour lactate clearance <10%. Compared with 10 patients with a 6-hour lactate clearance ≥10%, they presented a worse hepatosplanchnic perfusion as represented by significantly more severe derangements of ICG-PDR (9.7 (8–19) vs. 19.6 (9–32)%/min, *p* < 0.05) and pCO_2_ gap (33 (9.1-62) vs. 7.7 (3–58) mmHg, *p* < 0.05) at 6 hours. No other systemic, hemodynamic, metabolic, peripheral, or microcirculatory parameters differentiated these subgroups. We also found a significant correlation between ICG-PDR and pCO_2_ gap (*p* = 0.02).

**Conclusions:**

Impaired 6-hour lactate clearance could be associated with hepatosplanchnic hypoperfusion in some hyperdynamic septic shock patients. Improvement of systemic, metabolic, and peripheral perfusion parameters does not rule out the persistence of hepatosplanchnic hypoperfusion in this setting. Severe microcirculatory abnormalities can be detected in hyperdynamic septic shock patients, but their role on lactate clearance is unclear. ICG-PDR may be a useful tool to evaluate hepatosplanchnic perfusion in septic shock patients with persistent hyperlactatemia.

**Trial registration:**

ClinicalTrials.gov Identifier: NCT01271153

## Background

Several recent clinical studies have confirmed the strong prognostic value of hyperlactatemia in septic shock [1-3]. Both a single abnormal level and an impaired lactate clearance are related to morbidity and mortality [1-4]. Nguyen et al. demonstrated the relevance of lactate clearance in a study involving 111 septic patients [4]. Patients exhibiting a lactate clearance >10% after 6 hours of early resuscitation exhibited a significantly lower mortality than patients with <10% [4]. Furthermore, at least two randomized, controlled trials have explored lactate clearance as a potential resuscitation goal for septic shock patients with encouraging results [5,6].

The physiologic basis of lactate generation during shock has been matter of debate and research [7-11]. Hypovolemia-related hypoperfusion is probably the predominant pathogenic mechanism during the early pre-resuscitative phase. Some patients resolve sepsis-related circulatory dysfunction and clear lactate after initial fluid resuscitation, whereas others evolve into a persistent circulatory dysfunction with hyperlactatemia [12]. Although several potential hypoxic and nonhypoxic mechanisms have been associated with persistent hyperlactatemia [7-14], recent literature has highlighted the role of microcirculatory abnormalities [14] or hyperadrenergia [10,11,13] as the most likely determinants. This has occurred in parallel to a decline in the availability of gastric tonometry precluding clinicians to assess hepatosplanchnic perfusion in this setting. More importantly, the relative contribution of several potential factors to persistent hyperlactatemia after initial septic shock resuscitation has not been specifically addressed in comprehensive, clinical, physiological studies.

To address this subject, we designed an acute clinical physiological study to determine potential hemodynamic and perfusion-related parameters associated with 6-hour lactate clearance in a cohort of hyperdynamic septic shock patients with persistent hyperlactatemia. This pilot study evaluated several macrohemodynamic, metabolic, peripheral, hepatosplanchnic, and microcirculatory parameters immediately after preload optimization and 6 hours thereafter.

## Methods

This prospective study was conducted in a 16-bed, mixed, medical-surgical ICU at a university hospital in Santiago, Chile from September 2010 to December 2011. The local Institutional Review Board approved this study, and informed consent was obtained from each patient or surrogates. This study is part of an ongoing, randomized, double-blind, crossover, controlled trial exploring the acute effects of dobutamine on tissue hypoperfusion in hyperdynamic septic shock patients.

### Patient selection

All consecutive adult patients (>18 years) admitted to the ICU within 24 hours of onset of septic shock were considered eligible for this protocol. Specific inclusion criteria were: 1) septic shock according to the 2001 Consensus Definition (septic-related volume-refractory hypotension requiring vasopressors to maintain a mean arterial pressure (MAP) >65 mmHg) [15]; 2) persistent hyperlactatemia (arterial lactate >2.0 mmol/l after initial fluid loading); 3) cardiac index ≥2.5 l/min/m^2^; 4) sinus rhythm; and 5) mechanical ventilation and pulmonary artery catheter in place.

Patients were excluded according to the following criteria: 1) pregnancy; 2) anticipated surgery or dialytic procedure during the study period; 3) Child B or C liver cirrhosis; 4) do-not-resuscitate status or life expectancy less than 24 hours; and 5) preexisting conditions precluding peripheral perfusion assessment, such as hypothermia, Raynaud's disease, or severe peripheral vascular disease.

### General management of hyperdynamic septic shock patients

Recruited patients were resuscitated with a local norepinephrine (NE)-based, perfusion-oriented management protocol [16,17]. All patients were subjected to early aggressive source control. The main endpoint of ICU resuscitation was lactate normalization. Initial fluid resuscitation was directed at correcting basic hemodynamic parameters. NE was started and titrated to a MAP >65 mmHg in patients with persistent hypotension after fluid loading. Early intubation and mechanical ventilation were indicated for oxygen consumption reduction in patients with progressive hyperlactatemia or increasing NE requirements. Mechanical ventilation and sedation were managed in accordance with current protective strategies [18]. Cardiac index and related parameters were evaluated with a pulmonary artery catheter. Intravascular volume status was optimized following pulse pressure variation criteria [19]. Red blood cell transfusions were prescribed as necessary to maintain hemoglobin levels ≥8 g/dL. High-volume hemofiltration was used as a rescue therapy in refractory patients [17].

### Study protocol

Total study period was 6 hours. Two sets of hemodynamic and perfusion assessments were considered for the purpose of this specific study. A baseline set was performed immediately after preload optimization in the ICU as defined by a pulse pressure variation <10%. The second set was performed 6 hours thereafter.

Specific measured parameters at each time point were:

1. Macrohemodynamic parameters: MAP, heart rate, NE dose, pulse pressure variation (%), and pulmonary artery catheter-derived values.

2. Metabolic-related perfusion parameters: mixed venous O_2_ saturation (SvO_2_), arterial lactate (Radiometer ABL 735, Copenhagen Denmark), and mixed venous to arterial pCO_2_ gradient (p(cv-a)CO_2_). Six-hour lactate clearance was defined as the percent change in lactate level after 6 hours from the baseline measurement. It was calculated by using the following formula: baseline ICU lactate (hour 0) minus lactate at hour 6, divided by baseline ICU lactate, then multiplied by 100.

3. Peripheral perfusion parameters: a) Central to toe temperature gradient (Tc-toe), where central temperature was obtained from the pulmonary artery catheter, and toe temperature from the ventral face of the great toe of the right foot. A difference up to 7°C was considered normal. All skin temperatures were obtained with skin probes (HP 21078A, Hong Kong Kabeil Technology, Guangdong, China); and b) capillary refill time (CRT), measured by applying firm pressure to the distal phalanx of the index finger for 10 seconds. A chronometer recorded the time for return of the normal color at the ventral surface, and 4.0 seconds was defined as the upper normal limit.

4. Near-infrared spectroscopy (NIRS)-derived parameters [20]: Tissue oxygen saturation (StO_2_) was measured by a tissue spectrometer (InSpectra Model 325, Hutchinson Technology, MN, USA). The NIRS probe was placed on the skin of the thenar eminence and a sphygmomanometer cuff was wrapped around the arm over the brachial artery. After a 3-minute period to stabilize the NIRS signal, a vascular occlusion test (VOT) was performed. Arterial inflow was stopped by inflating the cuff to 50 mmHg above the systolic arterial pressure. After 3 minutes of ischemia cuff pressure was released and StO_2_ recorded continuously for another 3-minute period (reperfusion period). Baseline StO_2_ before VOT was recorded. During the reperfusion phase, the recovery slope of the StO_2_ signal was registered (expressed in percent per second).

5. Microcirculatory-derived parameters [21]: Sublingual microcirculation was assessed with sidestream dark field videomicroscopy imaging obtained with a 5x lens (Microscan® for NTSC, Microvision Medical, Amsterdam, NL). At each assessment, at least five 10–20-second video images were recorded. Image acquisition and analysis were performed following recent recommendations of a consensus conference [21]. A trained investigator performed image analysis in all cases. Parameters considered for this study were proportion of perfused vessels, perfused vessel density (PVD), and microcirculatory flow index (MFI).

6. Hepatosplanchnic-related perfusion parameters: a) gastric tonometry calculating the gastric-to-arterial pCO_2_ gradient (pCO_2_ gap) with a upper normal limit of 8 mmHg (Tonocap, Datex-Ohmeda Division, Helsinki, Finland) [22]; and b) plasma disappearance rate of indocyanine green (ICG-PDR) as a dynamic test for the assessment of liver function and global hepatosplanchnic blood flow [23]. The ICG-PDR was assessed with a noninvasive liver function monitoring system (LiMon, Pulsion Medical Systems, Munich, Germany). Each patient received an ICG finger clip that was connected to the liver function monitor. A dose of 0.25 mg/kg of ICG was injected through a central venous catheter. A range of ICG-PDR of 20-30%/min was considered normal.

### Statistical analysis

To address our objectives, we compared hemodynamic and perfusion-related parameters at baseline and at 6 hours in relation to lactate evolution. Specifically, we evaluated changes of hemodynamic and perfusion parameters in patients with a 6-hour lactate clearance ≥10% compared with those <10%.

Nonparametric statistics were performed, including Wilcoxon test for paired measurements and Mann–Whitney test for independent measurements. Spearman correlation was used to explore the relationship between ICG-PDR and pCO_2_ gap.

Results are expressed as median and range. A *p* value <0.05 was considered statistically significant. All reported *p* values are two-sided.

## Results

Fifteen hyperdynamic septic shock patients were included. Baseline characteristics of each individual patient are shown in Table 1. Nine of 15 patients had an abdominal sepsis (six with peritonitis secondary to gastrointestinal perforation, two with infected pancreatitis, and one with nonocclusive colonic ischemia).

**Table 1:** Baseline characteristics of 15 hyperdynamic septic shock patients according to a 6-hour lactate clearance higher or lower than 10%

**Pt.**	**Age**	**Survival**	**Sepsis source**	**SOFA**	**APACHE II**	**MAP**	**MAP**	**∆PP**	**∆PP**	** NE**	**NE**	** IAP**	**IAP**	**Lactate*Basal***	**Lactate*End***	**CO**_**2**_**Gap*Basal***	**CO**_**2**_**Gap*End***	**PDR*Basal***	**PDR**	**MFI**	**MFI**
						***Basal***	***End***	***Basal***	***End***	***Basal***	***End***	***Basal***	***End***						***End***	***Basal***	***End***
	*Lactate clearance >10%*																				
1	26	No	Peritonitis	10	24	66	68	6	8	0.11	0.05	9	9	2.4	1.6	-	-	24.6	32	2.8	2.3
2	66	Yes	Unknown	9	24	76	68	2	2	0.21	0.17	11	10	3.4	2.8	6.1	7.1	28.2	27.8	2.5	2.9
3	83	Yes	Respiratory	10	19	78	77	0	0	0.1	0.05	13	11	4.5	1.5	2.1	58.7	13.3	18.1	1.4	1.8
4	56	Yes	Peritonitis	9	25	82	67	8	29	0.09	0.12	18	21	3	2	5.3	4.6	19.9	17.7	2.3	1.8
5	83	No	Peritonitis	8	23	72	65	5	5	0.26	0.45	4	8	19	16.2	0	0.1	-	-	2.2	1.9
6	97	Yes	Peritonitis	8	25	86	68	10	17	0.25	0.14	9	20	2.5	1.8	8.3	16.7	28	31.1	2.5	2
7	66	Yes	Peritonitis	8	23	66	68	6	8	0.43	0.7	16	9	3.1	2.5	4.7	2.3	20.8	19.6	1.4	1.9
8	67	Yes	Peritonitis	13	20	75	70	8	7	0.21	0.23	14	15	4.9	3.3	6.2	9.3	19	23.3	2.2	2.3
9	69	Yes	Skin	13	16	72	71	10	16	0.25	0.34	6	13	3.5	2.9	10.7	7.7	11.7	9	1.3	2.5
10	71	Yes	Peritonitis	15	42	73	75	8	6	0.3	0.46	10	11	6.2	5.4	5	18.1	-	-	1.2	1.3
	*Lactate clearance <10%*																				
11	76	Yes	Unknown	13	36	72	72	5	2	0.08	0.08	11	15	2.8	2.7	5.9	9.1	14.4	9.7	1.9	2.1
12	58	Yes	Skin	19	38	64	64	4	2	0.4	0.65	18	18	4.9	5.6	23.9	52.5	19.5	8.1	2.2	2.9
13	73	Yes	Peritonitis	7	25	110	69	3,4	2	0.1	0.06	12	13	4.2	3.8	8.1	12	19	19	1.5	1.5
14	67	Yes	Peritonitis	15	29	69	70	0	0	0.03	0.05	7	8	2.5	2.5	61.6	62.9	9.8	11.3	1.0	1.8
15	56	Yes	Respiratory	8	19	73	92	9	5	0.1	0.08	9	6	2.8	3.2	10.8	33	8.4	7.8	1.9	1.9

Pt, patient; Survival, hospital survival; APACHE II, Acute Physiology and Chronic Health Evaluation II; IAP, intra-abdominal pressure (mmHg); SOFA, Sequential Organ Failure Assessment; MAP, mean arterial pressure (mmHg), ∆PP, Pulse pressure variation (%), NE, norepinephrine requirements (mcg/kg/min); lactate, (mmol/l); MFI, microcirculatory flow index; CO_2_ gap, gastric/arterial pCO_2_ gradient (mmHg); PDR, indocyanine green plasma disappearance rate (%min).

Six-hour lactate clearance was ≥10% in ten patients and lower in five (16% (12.9-66) vs. 3.6% (−14.3 to 9), respectively; *p* < 0.05). During the protocol, both groups received the same amounts of fluids (6-hour lactate clearance ≥10%: 780 ± 520 ml vs. 6-hour lactate clearance <10%: 690 ± 380 ml; *p* = 0.8).

**Figure 1:**
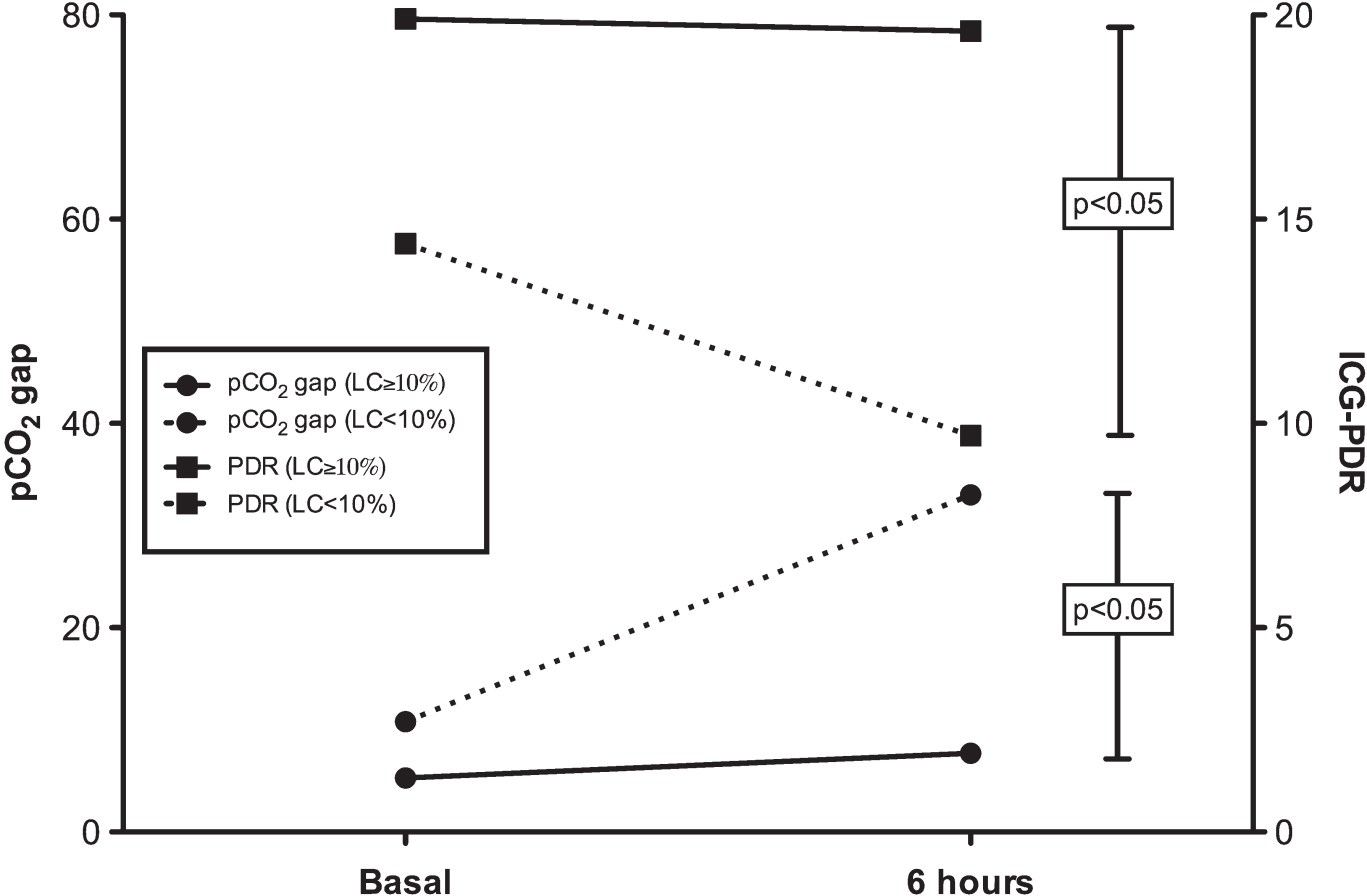
**Evolution of gastric-to-arterial pCO**_**2**_**gradients (pCO**_**2**_**gap in mmHg) and indocyanine green plasma disappearance rates (ICG-PDR in%/min) in patients exhibiting a 6-hour lactate clearance ≥ or <10%.** Patients with lower lactate clearance rates exhibited a significant increase in pCO_2_ gap and a decrease in ICG-PDR.

Compared with patients with a 6-hour lactate clearance ≥10%, patients with a lower lactate clearance presented a worse hepatosplanchnic perfusion as represented by significantly more severe derangements of ICG-PDR and pCO_2_ gap at 6 hours (Figure 1; Table 2). In the case of gastric pCO_2_ gap, this difference also was significant at baseline (Table 2). No other parameter differentiated these subgroups as shown in Table 2. When all data were pooled, a significant correlation between delta lactate (baseline lactate – final lactate) and delta ICG-PDR (*p* = 0.05, R2 = 0.3) was found.

**Table 2:** Multiparametric comparison between patients with a lactate clearance higher or lower than 10%

***Parameters***	**Lactate clearance ≥10% (n = 10)**			**Lactate clearance <10% (n = 5)**		
	***Basal***	***End***	***p*value**^***a***^	***Basal***	***End***	***p*value*#***
**Hemodynamic parameters**						
Pulse pressure variation (%)	7 (0–10)	7 (0–12)	*0.1*	4 (0–9)	2 (0–5)^b^	*0.07*
MAP (mmHg)	74 (66–86)	68 (65–77)	*0.12*	72 (64–90)	70 (64–90)	*0.9*
PAOP (mmHg)	14 (9–19)	11 (6–16)	*0.1*	16 (9–21)	13 (6–18)	*0.1*
CVP (mmHg)	13 (9–23)	13 (4–16)	*0.1*	13 (9–23)	12 (5–14)	*0.7*
Heart rate (bpm)	97 (80–119)	107 (65–132)	*0.12*	86 (74–120)	96 (74–136)	*0.1*
Cardiac index (l/min/m^2^)	3.4 (2.5-4.7)	4.1 (3–6.1)	*0.2*	3.1 (27–4.3)	4.2 (2.6-6.2)	*0.1*
APP (mmHg)	66 (54–99)	58 (48–64)	*0.3*	62 (50–98)	62 (48–86)	*0.6*
**Metabolic parameters**						
PaCO2 (mmHg)	35 (30–39)	36 (30–38)	*0.9*	35 (28–40)	35 (31–39)	*0.6*
SvO2 (%)	77 (65–91)	77 (71–94)	*0.2*	75 (66–79)	73 (68–79)	*0.07*
P(cv-a)pCO_2_ (mmHg)	4.3 (0.7-7)	3.5 (0.2-6)	*0.1*	6.3 (0.8-7.7)	3.5 (1–3.8)	*0.1*
**Peripheral perfusion parameters**						
CRT (s)	3.5 (1–7)	4 (2–6)	*0.6*	5 (1–12)	3 (1–12)	*0.9*
Central-peripheral temp. difference (°C)	8.6 (5.2-12.2)	6.5 (4–11.2)	*0.06*	11.8 (2.6-14)	10.5 (2.6-15.2)	*0.7*
**NIRS-derived parameters**						
StO_2_ (%)	84 (55–94)	85 (77–95)	*0.03*	74 (72–94)	82 (69–95)	*0.5*
StO_2_ recovery slope (%/s)	1.71 (0.4-4)	2.35 (0.8-3.4)	*0.3*	2.3 (0.4-7.6)	0.9 (0.8-5.1)	*0.7*
**Microcirculatory parameters**						
MFI (score)	2.1 (1.3-2.8)	1.95 (1.8-2.9)	*0.9*	1.87 (1–2.2)	1.92 (1.5-2.9)	*0.06*
Proportion of perfused vessels (%)	78.9 (62–93)	74.9 (67–99)	*0.7*	73.9 (40.8-82)	82.5 (71–92.1)	*0.1*
PVD (n/mm)	8.7 (7.5-10.7)	9.1 (6–11.3)	*0.9*	7.9 (4.1-10.4)	9 (7.2-10.7)	*0.08*
**Splanchnic perfusion parameters**						
ICG-PDR (%/min)	19.9 (6.1-28)	19.6 (9–32)	*0.48*	14.4 (8.4-19)	9.7 (8–19)*	*0.6*
pCO_2_ gap (mmHg)	5.3 (2–10.7)	7.7 (3–58)	*0.2*	10.8 (5.9-61)*	33 (9.1-62)*	*0.04*

Data presented as median (range) unless otherwise indicated.

^a^Wilcoxon test for paired measurements.

^b^*p* < 0.05 by Mann–Whitney test for independent measurements comparing final values between both subgroups.

MAP, mean arterial pressure; PAOP, pulmonary artery occlusion pressure; CVP, central venous pressure; APP, abdominal perfusion pressure; SvO_2_, mixed venous oxygen saturation; p(cv-a)CO_2_, mixed venous to arterial pCO_2_ gradient; CRT, capillary refill time; NIRS, near-infrared spectroscopy; StO_2_, tissue oxygen saturation; MFI, microcirculatory flow index; PVD, perfused vessel density; ICG-PDR, indocyanine green plasma disappearance rate; pCO_2_ gap, gastric to arterial pCO_2_ gradient.

Liver-related parameters also were not different between patients with 6-hour lactate clearance ≥ vs. <10% (bilirubin 0.9 (0.2-1.7) vs. 1.8 (0.6-4.1) mg/dl, *p* = 0.3; prothrombin time 46 (11–86) vs. 46 (34–59)%, *p* = 0.9; SGOT 174 (10–92) vs. 48 (18–105) U/l, *p* = 0.4). Nine patients exhibited intra-abdominal hypertension, but no patient had an abdominal compartment syndrome. Intra-abdominal pressure was not significantly correlated with lactate (*p* = 0.3), pCO_2_ gap (*p* = 0.3), neither with the ICG-PDR (*p* = 0.32).

**Figure 2:**
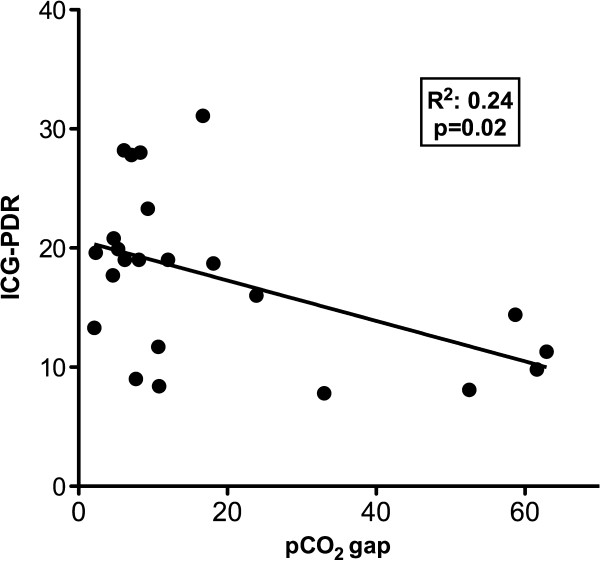
**Correlation between pooled indocyanine green plasma disappearance rate (ICG-PDR) and gastric-to-arterial pCO**_**2**_**gradient (pCO**_**2**_**gap) values.**

We found a mild but significant correlation between pooled ICG-PDR and pCO_2_ gap values (Figure 2).

## Discussion

Our main finding was the association of delayed 6-hour lactate clearance to hepatosplanchnic hypoperfusion but not to systemic hemodynamics or other perfusion parameters in hyperdynamic septic shock patients undergoing aggressive resuscitation.

The relevance of the concept of lactate clearance has been recently highlighted [4-6,24]. Therefore, it appears as critical to gain insight into potential pathogenic mechanisms associated to failure to clear lactate, especially in hyperdynamic septic shock patients in whom a low flow state is by definition a less probable mechanism. However, this task may be particularly complex in the setting of septic shock where an easy assumption to make is to attribute persistent hyperlactatemia to inadequate resuscitation. Indeed, although hypoperfusion is the most common cause of hyperlactatemia, increasing evidence for nonhypoxic related mechanisms, such as sustained hyperadrenergia with accelerated aerobic glycolysis, has recently expanded our understanding of the physiological meaning of lactate in sepsis [7-13]. Nevertheless, a limited number of factors, such as persistent global hypoperfusion, microcirculatory dysfunction, impaired hepatosplanchnic perfusion, or liver dysfunction, are more likely involved in a resuscitation scenario. A multimodal perfusion monitoring approach relying on relatively simple or noninvasive clinical tools can identify persistent global hypoperfusion. In our series of hyperdynamic septic shock patients, evolution of metabolic and peripheral perfusion parameters tends to rule out global hypoperfusion as a cause of impaired lactate clearance. In fact, these patients presented a median cardiac index >4 l/min/m2, SvO_2_ >70%, p(v-a)CO_2_ <5 mmHg, CRT <4 sec, and a thenar StO_2_ >80%, which is consistent with an hyperdynamic state.

Videomicroscopic bedside techniques have allowed direct visualization of microvascular flow, especially at the sublingual mucosa [25]. Microcirculatory flow or density abnormalities have been described and linked to bad outcome during septic shock [26,27]. Microcirculatory dysfunction with microvascular shunting could contribute to the genesis of hyperlactatemia in selected patients [12]. We recently demonstrated a significant correlation between hyperlactatemia and microcirculatory derangements in a large series of septic shock patients [14]. Interestingly, the majority of our patients exhibited significant microcirculatory abnormalities, irrespective of the status of lactate clearance. The median 6-hour values of proportion of perfused vessels, MFI, and PVD among microcirculatory parameters are well beyond the thresholds associated with morbidity or mortality in previous studies [1,2]. This is consistent with a profoundly abnormal StO_2_ recovery slope after VOT in our patients with a low lactate clearance, suggesting a persistent microvascular dysfunction.

Splanchnic contribution to hyperlactatemia may be secondary to an increased hypoxic or nonhypoxic gut lactate production, a decreased hepatic lactate clearance secondary to hypoperfusion or dysfunction, or a combination of both [28-31]. In our study, patients with a lactate clearance <10%, increased pCO_2_ gap, and decreased ICG-PDR to extremely abnormal values (medians of 33 mmHg and 9.7%, respectively) after 6 hours of resuscitation, suggesting the presence of sustained hepatosplanchnic hypoperfusion despite a global hyperdynamic flow status. Moreover, because no difference in liver enzymes between these subgroups was found, it is highly probable that alterations in hepatic flow rather than function were responsible for the low ICG-PDR. Our results confirm and expand previous findings by Friedman et al. that demonstrate a significant association of persistent hyperlactatemia at 24 hours with gastric mucosa hypoperfusion, but without correlation to systemic hemodynamics [32]. Our study, using a more comprehensive and multimodal monitoring approach, supports a relationship between persistent hyperlactatemia and hepatosplanchnic hypoperfusion; the latter is confirmed by an independent technique, such as ICG-PDR.

Several factors theoretically could have contributed to the development of both gut hypoperfusion and hyperlactatemia in our patients. An ischemic bowel may be a source of anaerobic lactate production, which depending on hepatic clearance could result in systemic hyperlactatemia, but only one of these patients was operated on for a nonocclusive colonic ischemia with secondary peritonitis. Some clinical reports have emphasized that intra-abdominal hypertension could induce hyperlactatemia through gut mucosal hypoperfusion and also decrease hepatic lactate clearance [33,34]. However, no difference in intra-abdominal hypertension between subgroups was observed in our series. Hypovolemia or high doses of vasoconstrictors could hasten hepatosplanchnic hypoperfusion, but this was not the case for our patients, because final pulse pressure variation was 2% (range, 0–5%), with NE doses of only 0.08 (0.05-0.6) mcg/kg/min.

The presence of occult hepatosplanchnic hypoperfusion in the setting of a global hyperdynamic state and with parallel improvements or no change in peripheral perfusion, metabolic, and microcirculatory parameters may appear contradictory. However, regional hepatosplanchnic vasoconstriction is a physiologic and early neurohumoral response to shock that can cause prolonged hypoperfusion and gut ischemia. Persistence of this state as represented by an abnormal gastric tonometry at 24 hours has a strong prognostic significance, irrespective of the status of systemic hemodynamics [35]. Unfortunately, a decline in the availability of gastric tonometry has precluded intensivists to use this valuable physiological monitor. Rather novel techniques, such as transcutaneous assessment of indocyanine green plasma disappearance rate, have become available, offering an opportunity for the early diagnosis of hepatic dysfunction or hypoperfusion [23]. Previous studies demonstrated a strong association between ICG-PDR and outcome in critically ill patients [36,37]. The ICG-PDR is influenced both by liver function and perfusion, but when evaluated in short periods of time, it mainly reflects hepatosplanchnic perfusion, because the function of liver cells does not change rapidly [38]. Thus, this method could eventually be used as a surrogate for gastric tonometry, and indeed we found a significant correlation between both techniques (Figure 2).

Our study has several limitations. Because it was designed as an acute clinical physiological pilot study, it included a limited number of patients, thus making it difficult to generalize conclusions. Only two sets of measurements were considered, thus precluding the possibility of capturing earlier or late changes of some of these parameters. The majority of patients were of an abdominal source, a factor that eventually could influence the results of the hepatosplanchnic perfusion assessment. We did not address potential nonhypoxic causes for persistent hyperlactatemia. Despite these limitations, we think that our data may be useful to reconsider the approach to persistent hyperlactatemia in hyperdynamic septic shock patients.

## Conclusions

Impaired 6-hour lactate clearance could be associated with hepatosplanchnic hypoperfusion in some hyperdynamic septic shock patients subjected to aggressive early resuscitation. An improvement in systemic, metabolic, and peripheral perfusion parameters does not rule out the persistence of hepatosplanchnic hypoperfusion. Relatively new techniques, such as noninvasive assessment of ICG plasma disappearance rate, could aid in interpreting a persistent hyperlactatemia. Severe microcirculatory abnormalities can be detected in hyperdynamic septic shock patients, but their role on lactate clearance is unclear. Future clinical studies should determine the best strategy to address persistent hyperlactatemia in hyperdynamic septic shock patients. Evaluation of hepatosplanchnic perfusion with current or developing technologies should probably be considered as a useful complementary tool in this setting.

## Abbreviations

APACHE: Acute Physiology and Chronic Health Evaluation; CRT: capillary refill time; IAP: intra-abdominal pressure; ICU: intensive care unit; MAP: mean arterial pressure; MFI: microcirculatory flow index; NE: norepinephrine; NIRS: near-infrared spectroscopy; pCO_2_ gap: gastric to arterial pCO_2_ gradient.; P(v-a)CO_2_: mixed venous to arterial pCO_2_ gradient; PVD: perfused vascular density; ICG-PDR: indocyanine green plasma disappearance rate; SOFA: Sequential Organ Failure Assessment; StO_2_: tissue oxygen saturation; SvO_2_: mixed venous oxygen saturation.

## Competing interests

The authors declare that they have no competing interests.

## Authors’ contributions

GH conceived the study, participated in its design and coordination, and helped to draft the manuscript. TR conceived the study, participated in its design and coordination, and helped to draft the manuscript. AB conceived the study, participated in its design and coordination, and helped to draft the manuscript. RC and JF helped to draft the manuscript and performed statistical analyses. AF analyzed microcirculatory images. EV performed sublingual microcirculation assessment. MR participated in its coordination and recruited patients. DB recruited patients. CM recruited patients. EK recruited patients. CI conceived the study and helped to draft the manuscript. All authors read and approved the final manuscript.
